# Clinical and inflammatory predictors of ICU admission and mortality in COVID-19: A retrospective multi-center cohort study from Saudi Arabia

**DOI:** 10.1097/MD.0000000000046716

**Published:** 2026-01-02

**Authors:** Zahraa Alali, Jumana Aljishi, Fatimah Saud Alzaher, Raha Al Dhafir, Muna Ali Alali, Fatemah Hussain Benalshaikh, Hani Makky Al Salam, Maryam Alhashim, Abdul Salam, Salma A. Aalmri, Kholoud Alwosaibai

**Affiliations:** a Department of Clinical Laboratory Sciences, College of Applied Medical Sciences, University of Hafr Al Batin, Hafr Al Batin, Saudi Arabia; b Internal Medicine Department, Qatif Central Hospital, Eastern Health Cluster, Qatif, Saudi Arabia; c Endocrinology Department, King Fahad University Hospital, Al Khobar, Saudi Arabia; d Allied Health Department, Eastern Health Cluster, Dammam, Saudi Arabia; e Nursing Department, Mohammed Al-Mana College for Medical Sciences, Dammam, Saudi Arabia; f Telemedicine Care, Eastern Health Cluster, Dammam, Saudi Arabia; g Department of Radiology, Qatif Central Hospital, Qatif, Saudi Arabia; h Radiology Department, Medical Imaging Services Center, King Fahad Specialist Hospital, Eastern Health Cluster, Dammam, Saudi Arabia; i Epidemiology and Biostatistics Administration, Population Health Management, King Fahad Specialist Hospital-Dammam, Eastern Health Cluster, Dammam, Saudi Arabia; j Biomedical Research Department, Research Center, King Fahad Specialist Hospital-Dammam, Eastern Health Cluster, Dammam, Saudi Arabia.

**Keywords:** COVID-19, cytokine storm, IL-8, intensive care unit, mortality, TNF-α

## Abstract

The early variant era of the coronavirus disease 2019 (COVID-19) pandemic was characterized by the emergence of new viral strains and limited vaccination coverage. Data describing the clinical, radiological, and inflammatory characteristics of affected patients in the Middle East remain scarce. This study aimed to fill this gap by comparing the clinical profiles, cytokine responses, and radiological patterns of intensive care unit (ICU) and non-ICU COVID-19 patients during the early phase of the pandemic in Saudi Arabia. A retrospective cohort study was conducted between February and August 2021 across 3 hospitals in the Eastern Province of Saudi Arabia. A total of 117 polymerase chain reaction-confirmed COVID-19 patients were included and categorized into ICU (n = 56) and non-ICU (n = 61) groups. Demographic characteristics, comorbidities, radiological findings, and serum levels of interleukin (IL)-6, IL-8, and tumor necrosis factor-alpha (TNF-α) were analyzed. Logistic regression identified predictors of ICU admission, while Cox regression evaluated mortality risk factors. ICU patients were older and more symptomatic, particularly with shortness of breath and chest pain. Older age, comorbidities, and elevated IL-8 and TNF-α independently predicted ICU admission, while IL-8 and TNF-α were associated with increased mortality. Amikacin use correlated with higher mortality (hazard ratio: 5.12, *P* = .015). Radiologically, 86% of ICU patients exhibited bilateral peripheral infiltrates. Distinct inflammatory and radiological profiles characterized severe COVID-19 cases during the early variant era. Routine cytokine profiling at hospital admission may aid clinical triage, while future studies should validate these biomarkers as prognostic tools in pandemic preparedness.

## 1. Introduction

Coronavirus disease 2019 (COVID-19) has resulted in a high rate of hospitalizations and intensive care unit (ICU) admissions worldwide, with severe cases frequently progressing to acute respiratory distress syndrome and multi-organ failure, significantly increasing the risk of mortality.^[1–4]^ The severity of COVID-19 is strongly linked to an excessive immune response, commonly referred to as a cytokine storm, which contributes to widespread inflammation, tissue damage, and organ failure.^[5,6]^ In clinical practice, ICU admission often reflects the severe spectrum of COVID-19 characterized by respiratory failure and systemic hyperinflammation. Therefore, comparing ICU and non-ICU patients provides a pragmatic framework to explore how inflammatory responses, including cytokine storm phenomena, correlate with disease severity and clinical outcomes.

Multiple studies from different regions, including the United States, Malaysia, and Germany, have demonstrated that pro-inflammatory cytokines such as interleukin (IL)-6, IL-8, tumor necrosis factor-alpha (TNF-α), IL-2, and IL-10, along with chemokines including CXCL1, CXCL2, CXCL5, CXCL8, and CXCL10, play a pivotal role in disease progression and mortality among ICU-admitted patients. These inflammatory mediators exacerbate lung injury and are associated with worse clinical outcomes.^[7–9]^ Furthermore, radiological findings in critically ill COVID-19 patients frequently reveal bilateral ground-glass opacities and alveolar consolidation, which are indicators of severe lung involvement.^[10,11]^ Studies reported that high admission levels of these cytokines independently predicted mortality in hospitalized patients. Understanding this relationship is critical for clinical management, as early identification of patients with marked cytokine elevation may facilitate timely interventions such as targeted antiinflammatory therapy, escalation of care, and closer monitoring (ref).

However, the specific clinical and immunological factors influencing disease progression between ICU and non-ICU patients remain unclear in Saudi Arabia, particularly during the early variant era when vaccination coverage was limited, and treatment protocols were still evolving. Regional differences in patient demographics, healthcare systems, and therapeutic practices may influence disease trajectories and outcomes. Saudi Arabia, with its unique population structure and evolving healthcare responses to the pandemic, provides an important setting for documenting COVID-19 severity patterns during this transitional period. Understanding how inflammatory markers and radiographic progression correlate with clinical deterioration in this context is crucial for improving patient care and preparedness for future outbreaks.

This study focused on the early phase of the COVID-19 pandemic because it represented a period when vaccination coverage was minimal and therapeutic protocols were still evolving. Studying this period provides insight into the unmodified immune and clinical response to severe acute respiratory syndrome coronavirus 2 (SARS-CoV-2) infection, allowing clearer interpretation of cytokine dynamics without confounding from widespread immunization or targeted immunotherapy. Given these considerations, this multi-center retrospective study, conducted in Saudi Arabia between February and August 2021, aims to compare the clinical, radiological, and immunological characteristics of ICU-admitted and non-ICU COVID-19 patients during the early variant era. By characterizing differences in cytokine levels, radiographic features, and clinical outcomes, this study provides region-specific insights into severe COVID-19 during a pivotal stage of the pandemic.

## 2. Methods

### 2.1. Patient recruitment and study design

We retrospectively analyzed 117 polymerase chain reaction (PCR)-confirmed COVID-19 cases, including 56 ICU patients and 61 non-ICU patients, collecting data on demographics, comorbidities, clinical symptoms, cytokine levels (IL-6, IL-8, TNF-α), radiological findings, and treatment regimens. Patients were initially admitted or seen at the participating hospitals between February and August 2021. All consecutive patients with PCR-confirmed COVID-19 who met the inclusion criteria during the study period were included. Because this was a retrospective review, the final sample of 117 patients therefore represents a convenience sample comprising all eligible cases admitted to the 3 participating hospitals between February and August 2021. To ensure representation of the diverse population, patient data were collected from 3 major regional hospitals: Dammam Central Hospital, Qatif General Hospital, and Babtain Hospital. These hospitals were selected because they served as major referral centers for COVID-19 cases in the Eastern Province during the early-pandemic phase. Dammam Central and Qatif General Hospitals provided tertiary care and managed a high volume of moderate-to-severe cases, while Babtain Hospital functioned as a specialized facility for cardiopulmonary and critical care services.

Inclusion criteria were: adults aged ≥ 18 years; confirmed SARS-CoV-2 infection by RT-PCR; and hospital admission to one of the 3 participating centers between February and August 2021. Both ICU and non-ICU patients were included to capture a wide range of disease severity. Exclusion criteria included: patients with incomplete clinical or laboratory data.

No matching procedures were performed between ICU and non-ICU groups. The 2 cohorts were compared as observed, based on their actual clinical and demographic characteristics, to reflect real-world differences in disease severity and patient profiles.

Demographic and clinical data were retrieved from the Waqaia database, electronic medical records, and structured patient questionnaires. Upon hospital admission, comprehensive data on patient characteristics, comorbidities, clinical symptoms, and disease progression were documented. Patients were systematically monitored throughout hospitalization, with outcomes recorded as either recovery or mortality. ICU admission status was identified from hospital electronic medical records and confirmed through the Waqaia national COVID-19 database, which records all hospitalized cases and their level of care. Each patient’s admission and discharge notes, along with bed allocation records, were reviewed to verify whether the patient required ICU-level management during hospitalization. Patients were followed prospectively during hospitalization from the date of 1st evaluation until discharge or in-hospital death, whichever occurred 1st. Surviving patients were censored at discharge.

### 2.2. Ethical approval

This study was approved by the Institutional Review Board affiliated with King Fahad Specialist Hospital-Dammam under protocol #BMR002. All samples and data were collected in accordance with ethical guidelines, ensuring patient confidentiality. Informed consent was obtained from all patients enrolled in the study.

### 2.3. Sample collection and processing

All participating hospitals followed standard national protocols for the clinical management of COVID-19 during the early-pandemic period. Upon admission, all PCR-confirmed patients underwent baseline clinical evaluation, including complete blood count, and chest imaging. Blood samples for cytokine analysis were typically collected within the 1st 24 to 48 hours of admission, coinciding with initial laboratory work-up. Additional samples were obtained during the course of hospitalization at the discretion of the treating physician if clinical deterioration occurred. Peripheral blood samples (3–5 mL) were collected from all patients following standard venipuncture protocols. Serum was separated by centrifugation at 2500 rpm for 15 minutes and stored at −80°C until further immunological analysis.

Chest X-rays were routinely performed at admission and repeated during the ICU stay, typically within 2 to 5 days based on clinical condition, with a final image obtained at discharge or the end of ICU care. Radiological assessment primarily involved serial chest X-rays for all admitted patients. Chest computed tomography was reserved for cases with diagnostic uncertainty or clinical worsening. ICU admission criteria followed institutional guidelines, typically triggered by respiratory distress, oxygen saturation < 90% on high-flow oxygen, or hemodynamic instability. Discharge from ICU occurred when patients achieved clinical stability and no longer required advanced respiratory or hemodynamic support.

### 2.4. Cytokines quantification

Serum cytokine levels, including IL-6, IL-8, and TNF-α, were measured using commercially available enzyme-linked immunosorbent assay (ELISA; Bio‐Plex Pro Human Cytokine 8‐plex Assay [Cat. No.M50000007A]; Bio‐Rad Laboratories, Hercules) kits, following the manufacturers’ protocols. Assays were performed in duplicate, and absorbance was read using a microplate reader to ensure accuracy and reproducibility. These cytokines were selected because IL-6, IL-8, and TNF-α are key pro-inflammatory mediators consistently implicated in COVID-19-related cytokine storm and disease severity.

### 2.5. Statistical analysis

Categorical variables were summarized using counts and percentages, while continuous variables were reported as means with standard deviations. Group comparisons between ICU and non-ICU patients were performed using independent *t* tests for continuous variables and chi-square tests for categorical variables. Comparisons of cytokines concentration between ICU and non-ICU patients were analyzed using the A Mann–Whitney *U* test (non-parametric test). The study focused on 2 key outcomes: ICU admission and mortality. To identify factors associated with ICU admission, univariate logistic regression models were applied. For the logistic regression analysis, the dependent variable was ICU admission (yes/no). Independent variables included age, sex, presence of chronic disease, hypertension, diabetes, and cytokine levels (IL-6, IL-8, and TNF-α). For the mortality outcome, survival time was calculated from the date of COVID-19 diagnosis to either the date of death or the last follow-up. Univariate Cox proportional hazards models were used to explore potential predictors of mortality. For the Cox proportional hazards model, the dependent variable was mortality during hospitalization. Independent variables included age, sex, comorbidities (chronic disease, hypertension, diabetes, other diseases), cytokine levels (IL-6, IL-8, and TNF-α), and medication use (amikacin, ceftriaxone, azithromycin, enoxaparin, dexamethasone, and meropenem). All statistical analyses were carried out using RStudio (Integrated Development Environment for R, Version 2025; Posit Software, PBC, Boston), with *P*-values below .05 considered statistically significant.

## 3. Results

### 3.1. Clinical and demographic characteristics of COVID-19 patients

In this study, involving a total of 117 COVID-19 patients, 56 (47.9%) were admitted to the ICU, while 61 (52.1%) were managed in non-ICU settings (Table 1). Notably, ICU patients exhibited a significantly higher mean age (55.73 ± 14.1) compared to non-ICU patients (36.21 ± 9.8) (*P* = .006). Gender distribution varied significantly between the 2 groups (*P* = .014), with 55.4% of ICU patients being male, compared to 77.0% in the non-ICU group (Table 1).

**Table 1:** Baseline demographic and clinical characteristics of COVID-19 patients admitted to ICU and non-ICU wards in 3 Eastern Province Hospitals, Saudi Arabia.

Characteristics	ICU (severe) (n = 56)	Non-ICU (mild) (n = 61)	*P*-value
Baseline characteristics			
Age (mean ± SD)	55.73 ± 14.1	36.21 ± 9.8	.006*
Gender – male	31 (55.4)	47 (77)	.014*
Symptoms			
Showing symptoms (n, %)	56 (100)	12 (19.7)	<.001*
Shortness of breath (n, %)	34 (60.7)	6 (9.8)	<.001*
Chest pain (n, %)	8 (14.3)	0 (0.0)	.001*
Cough (n, %)	22 (39.3)	20 (32.8)	.4
Fever (n, %)	32 (57.1)	18 (29.5)	.003*
Diarrhea (n, %)	8 (14.3)	4 (6.6)	.063*
Headache, fatigue, loss of appetite (n, %)	14 (25)	18 (29.5)	.17
Vomitting (n, %)	4 (7.1)	5 (8.2)	.1
Preexisting conditions			
Chronic disease (n, %)	17 (30.4)	11 (18)	.2
Hypertension (n, %)	10 (17.9)	7 (11.5)	.33
Diabetes (n, %)	14 (25)	8 (13.1)	.1
Non-communicable diseases (NCDs) (n, %)	22 (39.3)	5 (8.2)	<.001*

COVID-19 = coronavirus disease 2019, ICU = intensive care unit, SD = standard deviation.

* Statistically significant.

Clinically, all ICU patients presented with symptoms, whereas only 19.7 % of non-ICU patients were symptomatic (*P* < .001). Shortness of breath (60.7 % vs 9.8 %, *P* < .001), chest pain (14.3 % vs 0 %, *P* = .001), and fever (57.1 % vs 29.5 %, *P* = .003) were markedly more frequent among ICU admissions. Other symptoms, including cough, diarrhea, headache, fatigue, and vomiting, showed no significant differences between groups. (Table 1, Figure S1, Supplemental Digital Content, https://links.lww.com/MD/Q974).

Analysis of preexisting conditions revealed varying prevalence between ICU and non-ICU COVID-19 patients. Notably, chronic diseases were observed in 30.4% of ICU patients compared to 18.0% of non-ICU patients, though this difference did not reach statistical significance (*P* = .200). Similarly, hypertension was present in 17.9% of ICU patients and 11.5% of non-ICU patients, showing no significant difference (*P* = .328). Diabetes demonstrated a higher prevalence in ICU patients (25.0%) compared to non-ICU patients (13.1%) (*P* = .100). However, non-communicable diseases such as heart diseases, kidney diseases, or cancers exhibited a significant difference, with 39.3% prevalence in ICU patients compared to 8.2% in non-ICU patients, showing a highly significant association (*P* < .001) (Table 1, Figure S2, Supplemental Digital Content, https://links.lww.com/MD/Q974).

### 3.2. Inflammatory cytokine profiles in ICU and non-ICU patients

**Figure 1.**
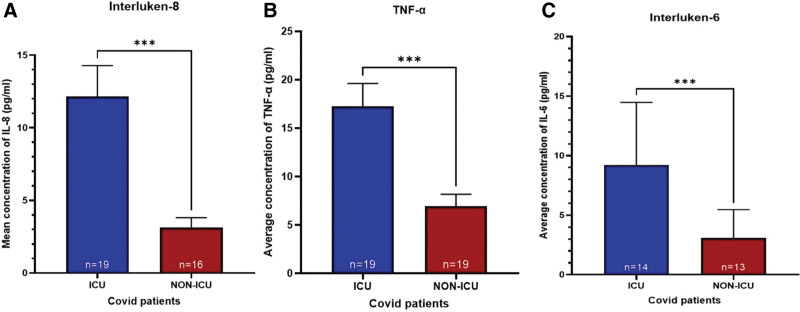
Comparison of pro-inflammatory cytokine levels in ICU and non-ICU COVID-19 patients. The bar graphs illustrate the mean concentrations (pg/mL) of interleukin-6 (IL-6), interleukin-8 (IL-8), and tumor necrosis factor-alpha (TNF-α) in ICU-admitted and non-ICU COVID-19 patients. (A) IL-8 levels are shown for ICU patients (n = 19, blue bars) and non-ICU patients (n = 16, red bars). (B) TNF-α levels are presented for ICU patients (n = 19, blue bars) and non-ICU patients (n = 19, red bars). (C) IL-6 levels are compared between ICU patients (n = 14, blue bars) and non-ICU patients (n = 13, red bars). A statistically significant difference (*P* < .001, Mann–Whitney *U* test [non-parametric test]) is observed in all cytokines, as indicated by triple asterisks (***). COVID-19 = coronavirus disease 2019, ICU = intensive care unit.

**Figure 2.**
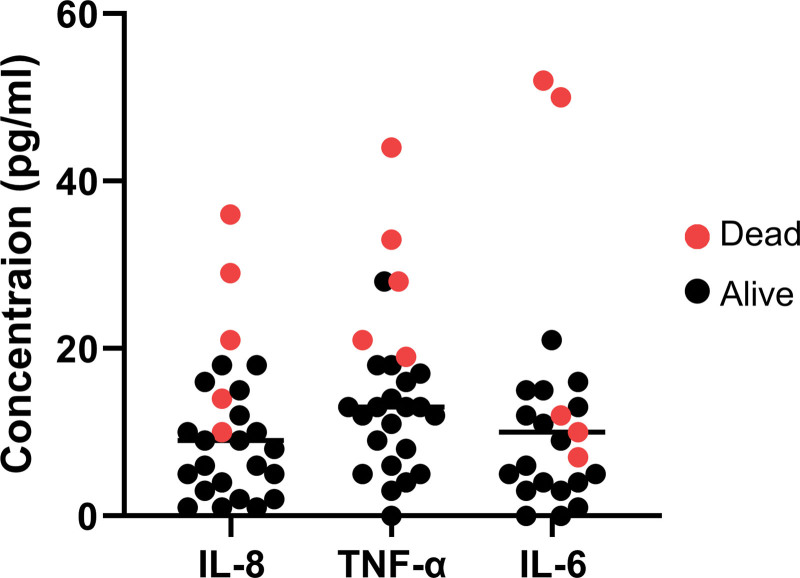
Pro-inflammatory cytokine levels in ICU patients. Dot plots showing the concentrations of IL-8, TNF-α, and IL-6 in ICU patients with COVID-19. Each dot represents an individual patient. Red dots indicate patients who died, while black dots represent survivors. Patients who died exhibited higher cytokine levels compared to survivors. COVID-19 = coronavirus disease 2019, ICU = intensive care unit, IL = interleukin, TNF-α = tumor necrosis factor-alpha.

ICU patients exhibited significantly elevated cytokine concentrations compared to their non-ICU counterparts. The levels IL-8 were significantly increased in the ICU group (11.75 pg/mL, 95% confidence interval [CI]: 6.28–17.22) relative to the non-ICU cohort (4.18 pg/mL, 95% CI: 2.38–5.98, *P* = .0006) (Fig. 1A). A comparable pattern was observed for TNF-α, with ICU patients exhibiting a mean concentration of 16.35 pg/mL (95% CI: 9.14–23.56), compared to 7.12 pg/mL (95% CI: 4.38–9.86, *P* = .0003) in non-ICU patients (Fig. 1B). Similarly, the mean IL-6 concentration in ICU patients was 31.26 pg/mL (95% CI: 14.71–47.81), significantly higher than in non-ICU patients, whose mean IL-6 was 5.94 pg/mL (95% CI: 2.23–9.65, *P* = .0004) (Fig. 1C). Further, we found that COVID-19 patients that admitted in ICU and died from the infection have high levels of pro-inflammatory cytokines compared to the patients that survived after the infection reflecting the heightened inflammatory response seen in severe cases during this pandemic phase (Fig. 2).

### 3.3. Logistic regression for predicting ICU admission

Logistic regression identified several factors associated with ICU admission among COVID-19 patients. Significant predictors included increasing age (OR 1.15, 95% CI: 1.09–1.21, *P* < .001), female sex (OR 2.38, 95% CI: 1.03–5.26, *P* = .041), the presence of other non-communicable diseases (OR 6.86, 95% CI: 2.37–19.84, *P* = .0004), elevated IL-8 (OR 1.21, 95% CI: 1.04–1.40, *P* = .013), and elevated TNF-α (OR 1.14, 95% CI: 1.02–1.27, *P* = .010). Among non-significant but clinically relevant variables, IL-6 showed a positive but non-significant trend (OR: 1.08, 95% CI: 0.99–1.18, *P* = .094), while chronic disease (OR: 1.90, *P* = .200), hypertension (OR: 1.69, *P* = .328), and diabetes (OR: 2.20, *P* = .100) all exhibited higher odds of ICU admission without reaching statistical significance (Table 2).

**Table 2:** Logistic regression analysis of demographic, clinical, and inflammatory covariates predicting ICU admission among COVID-19 patients in 3 Eastern Province Hospitals, Saudi Arabia.

Variable	Unit	Odds ratio (95% CI)	*P*-value
Age	–	1.15 (1.09–1.21)	<.001*
Gender	Female	Reference	
	Male	2.38 (1.03–5.26)	.041*
Chronic disease	No	Reference	
	Yes	1.94 (0.82–4.62)	.13
Hypertension	No	Reference	
	Yes	1.68 (0.59–4.76)	.33
Diabetes	No	Reference	
	Yes	2.21 (0.85–5.76)	.1
Other disease	No	Reference	
	Yes	6.86 (2.37–19.84)	.0004*
IL-6	–	1.08 (0.99–1.18)	.094
IL-8	–	1.21 (1.04–1.40)	.013*
TNF	–	1.14 (1.02–1.27)	.01*

CI = confidence interval, COVID-19 = coronavirus disease 2019, ICU = intensive care unit, IL = interleukin, TNF-α = tumor necrosis factor-alpha.

* Statistically significant.

### 3.4. Survival analysis for predicting mortality

Age was significantly associated with increased mortality risk (hazard ratio [HR]: 1.04, 95% CI: 1.01–1.07, *P* = .004), indicating that older patients were more likely to die. Gender, chronic disease, hypertension, diabetes, and other diseases were not significantly associated with mortality. Regarding inflammatory markers, higher TNF (HR 1.11, 95% CI: 1.01–1.21, *P* = .032) and IL-8 levels (HR 1.23, 95% CI: 1.03–1.46, *P* = .02) were significantly associated with increased mortality, showing that elevated cytokine levels correlated with higher death risk, whereas IL-6 levels were not (Table 3). In the Cox regression analysis for medication use (Table 4), only Amikacin showed a statistically significant association with increased mortality risk (HR 5.12, 95% CI: 1.37–19.15, *P* = .015). The other medications, including Ceftriaxone (HR 1.06, 95% CI: 0.38–2.95, *P* = .91), Azithromycin (HR 1.00, 95% CI: 0.36–2.80, *P* = .99), Enoxaparin (HR 0.62, 95% CI: 0.21–1.81, *P* = .38), Dexamethasone (HR 0.51, 95% CI: 0.16–1.62, *P* = .26), and Meropenem (HR 0.36, 95% CI: 0.05–2.80, *P* = .33) were not significantly associated with mortality (*P* > .05 for all). Despite the relatively high hazard ratios for some of these medications, none reached statistical significance.

**Table 3:** Cox regression analysis of demographic, clinical, and inflammatory covariates predicting mortality among COVID-19 patients admitted to ICU and non-ICU wards in 3 Eastern Province Hospitals, Saudi Arabia.

Variable		Hazard ratio (95% CI)	*P*-value
Age		1.04 (1.01–1.07)	.004*
Gender	Female	Reference	
	Male	0.62 (0.22–1.72)	.36
Chronic disease	No	Reference	
	Yes	1.42 (0.48–4.22)	.53
Hypertension	No	Reference	
	Yes	1.86 (0.38–9.00)	.44
Diabetes	No	Reference	
	Yes	1.23 (0.39–3.91)	.72
Other disease	No	Reference	
	Yes	0.93 (0.33–2.61)	.89
TNF		1.11 (1.01–1.21)	.032*
IL-6		0.99 (0.95–1.03)	0.63
IL-8		1.23 (1.03–1.46)	.02*

CI = confidence interval, COVID-19 = coronavirus disease 2019, ICU = intensive care unit, IL = interleukin, TNF-α = tumor necrosis factor-alpha.

* Statistically significant.

**Table 4:** Cox regression analysis of medication use as covariates predicting mortality among COVID-19 patients admitted to ICU and non-ICU wards in 3 Eastern Province Hospitals, Saudi Arabia.

Variable	Hazard ratio (95% CI)	*P*-value
Ceftriaxone	1.06 (0.38–2.95)	**.91**
Azithromycin	1.00 (0.36–2.80)	.99
Enoxaparin	0.62 (0.21–1.81)	.38
Dexamethasone	0.51 (0.16–1.62)	.26
Meropenem	0.36 (0.05–2.80)	.33
Colistin	2.64 (0.33–21.20)	.36
Tazocin	1.70 (0.60–4.83)	.32
Linzolid	1.95 (0.25–15.39)	.53
Vancomycin	1.89 (0.67–5.36)	**.23**
Amikicin	5.12 (1.37–19.15)	**.015** *

CI = confidence interval, COVID-19 = coronavirus disease 2019, ICU = intensive care unit.

* Statistically significant.

### 3.5. Radiological findings of ICU patients

Among 56 ICU-admitted COVID-19 patients, the most common radiological pattern was bilateral, peripheral, and lower-lobe infiltrates (48 patients, 86%), consistent with typical COVID-19 pneumonia. A smaller proportion showed indeterminate findings (7%), such as unilateral or focal opacities, while normal radiographs were observed in only 7% early in the disease course. As the disease progressed, all cases with initially normal or indeterminate findings evolved into the characteristic COVID-19 pattern (Table S1, Supplemental Digital Content, https://links.lww.com/MD/Q975).

## 4. Discussion

This study aimed to compare the clinical, radiological, and inflammatory profiles of ICU and non-ICU COVID-19 patients during the early phase of the pandemic in Saudi Arabia. ICU patients were older (mean = 55.7 ± 14.1 years) and more often male (55%) than non-ICU patients (36.2 ± 9.8 years; 77%). Respiratory symptoms such as shortness of breath (61% vs 10%) and fever (57% vs 30%) were significantly more common among ICU admissions. Radiologically, bilateral peripheral infiltrates with reduced lung volumes were the predominant findings (86%). Elevated TNF-α and IL-8 levels were strongly associated with disease severity and mortality. These findings collectively underscore the contribution of age, comorbidities, and cytokine-driven inflammation to severe COVID-19 outcomes.

It is well-documented that age is a consistent risk factor for adverse outcomes in severe COVID-19 cases,^[3,12]^ which verifies our findings. Additionally, there was a significant difference in gender distribution, with a greater proportion of males observed in the non-ICU group.^[12]^ This demographic variation underscores the importance of considering age and gender in the assessment of COVID-19 severity and prognosis. This aligns with prior studies showing older age as a major predictor of severe COVID-19, likely due to immune senescence, higher angiotensin-converting enzyme-2 expression, and greater prevalence of comorbidities. The higher odds of ICU admission observed among females in this cohort likely reflect residual confounding (age/comorbidity distribution) and early-pandemic selection patterns, rather than a sex-specific biological risk.

Presentation of symptoms played a crucial role in distinguishing between severe and mild cases of COVID-19. All ICU patients presented with symptoms, emphasizing the importance of attentive monitoring for individuals displaying clinical manifestations. Specific symptoms such as shortness of breath, chest pain, and fever were significantly more prevalent in ICU patients, highlighting their potential as early indicators of disease severity. This aligned with previous investigations showing that these symptoms are characteristic of severe COVID-19 cases and are associated with a higher disease burden, including the development of long-term health implications of the disease.^[13,14]^ Studies have consistently reported fever as one of the primary symptoms in severe COVID-19 cases requiring ICU admission.^[15]^ The predominance of dyspnea and fever among ICU patients mirrors global findings, reflecting the correlation between systemic inflammation and hypoxemic respiratory failure.

Preexisting conditions emerged as crucial determinants of COVID-19 outcomes. In our cohort, the prevalence of chronic diseases was higher among ICU patients compared to those in non-ICU settings. Specifically, cardiovascular, renal, and oncological comorbidities exhibited a significantly greater prevalence in ICU patients, highlighting the elevated risk associated with these conditions in severe COVID-19 cases. These findings align with previous studies indicating that patients with active cancer are prone to experiencing more severe COVID-19 outcomes, often requiring ICU admission.^[16,17]^ This heightened risk is particularly observed among the elderly and individuals with comorbidities such as cardiovascular disease, arterial hypertension, diabetes mellitus, chronic lung disease, renal failure, cerebrovascular disease, or malignancy.^[17]^ Similar to other regional and international cohorts, hypertension and diabetes were frequent among severe cases, suggesting that metabolic and cardiovascular dysregulation may amplify cytokine responses.

The cytokine analysis demonstrated significant elevation of the levels of IL-6, IL-8, and TNF-α in severe cases compared to mild cases. The significantly elevated concentration of pro-inflammatory cytokines observed in patients admitted to the ICU provides compelling evidence of a phenomenon known as a “Cytokine Storm.” This dysregulated immune response has been significantly associated with severe cases of COVID-19.^[18,19]^ In the context of COVID-19, an excessive release of pro-inflammatory cytokines is a result of host immune response overstimulation against SARS-CoV-2 infection, which leads to damage to healthy tissues and organs.^[20]^ The association between elevated IL-6, IL-8, and TNF-α concentrations and disease severity in ICU COVID-19 patients has been well-documented^[21]^ and was used to develop therapeutic and prognostic strategies to control the severity of the disease.^[22]^ IL-6 in particular has been implicated in the pathophysiology of acute respiratory failure in COVID-19, contributing to hyperinflammation and immune dysregulation.^[23]^ Likewise, IL-8 and TNF-α play key roles in COVID-19–related inflammation. IL-8 promotes lung injury by attracting neutrophils, while TNF-α drives endothelial activation and immune dysregulation.^[24,25]^ In the context of COVID-19, the significant elevation of IL-6, IL-8, and TNF-α in ICU patients (as demonstrated in a large cohort study by Del Valle et al^[26]^) strongly supports a pathophysiologic role for cytokine storm in driving severe disease. In Saudi Arabia, our findings align with several studies,^[27,28]^ including one by Alosaimi et al that examined the association between complement dysregulation and COVID-19 severity in 53 patients (27 mild, 26 critical). This study revealed significantly elevated levels of inflammatory cytokines (IL-1β, IL-6, IL-8, and TNF-α) in critical patients, which were correlated with in-hospital deaths among individuals with critical COVID-19.^[29]^ While IL-8 and TNF-α showed robust associations with ICU admission, non-significant trends for chronic disease may reflect limited power, diagnostic heterogeneity, and partial mediation through inflammatory activity.

Our survival analysis indicates that elevated TNF-α and IL-8 levels were significantly associated with increased mortality risk among COVID-19 patients, highlighting their potential as prognostic biomarkers. Patients with higher TNF-α and IL-8 concentrations had poorer survival, suggesting that an intense inflammatory response portends worse outcomes. These results are supported by prior studies: for example, a large prospective study found that high admission levels of IL-6, IL-8, and TNF-α were each significantly associated with mortality, independent of other risk factors.^[26]^ The prognostic significance of TNF-α and IL-8 in our study underscores that a hyper-inflammatory state (marked by these cytokines) is not only a marker of disease severity but also directly linked to survival odds. Clinically, this means TNF-α and IL-8 could serve as early predicting markers: patients exhibiting a cytokine profile with high TNF-α/IL-8 might warrant aggressive monitoring and intervention. Our findings contribute to the growing evidence that a specific cytokine “signature” – including IL-8 and TNF-α–can stratify patients by mortality risk, potentially guiding clinicians in identifying which patients are at greatest risk of deterioration.^[30]^ Given these findings, baseline and follow-up cytokine testing (particularly for IL-8 and TNF-α) at hospital admission and again within 5 to 7 days or upon clinical deterioration may assist in identifying patients at risk of cytokine storm. Elevated IL-8 (>10 pg/mL) or TNF-α (>15 pg/mL) should prompt closer monitoring and consideration of early antiinflammatory therapy in line with established treatment protocols.

Our analysis found no significant link between commonly used medications (Ceftriaxone, Azithromycin, Enoxaparin, and Dexamethasone) and mortality. Although Dexamethasone has been shown to reduce mortality in severe COVID-19 cases,^[31]^ this effect was not observed in our cohort, likely due to patient heterogeneity and unmeasured confounders, typical challenges in early-pandemic real-world datasets. In contrast, Amikacin was significantly associated with increased mortality (HR 5.12, *P* = .015), possibly due to its nephrotoxic and ototoxic effects,^[32]^ particularly in critically ill patients. Supporting this, a prospective study in a Sub-Saharan African ICU identified Amikacin as an independent risk factor for acute kidney injury.^[33]^ These findings emphasize the need for cautious use of Amikacin and strict renal function monitoring, especially in ICU settings. While our exploratory, unadjusted analyses did not demonstrate a mortality benefit for dexamethasone or other agents, this contrasts with large randomized trials reporting benefit for corticosteroids, IL-6, and JAK inhibitors in severe COVID-19 (Ref). Discrepancies likely reflect limited power, residual confounding, and heterogeneity in timing and dosing during early 2021, whereas the amikacin signal may relate to confounding by indication and the known nephrotoxicity risk in critically ill patients.

The chest X-ray findings in our ICU cohort (86% with bilateral, peripheral lung involvement that evolved over time) are in line with the characteristic imaging patterns reported in COVID-19 pneumonia. Bilateral patchy opacities with a peripheral distribution are a hallmark of COVID-19 lung involvement. For instance, nearly 90% of patients in 1 large series showed a peripheral predominance of infiltrates on initial radiographs, and over 60% had bilateral disease.^[34]^ In our critically ill patients, the predominance of bilateral peripheral consolidation likely reflects advanced diffuse pneumonia consistent with acute respiratory distress syndrome. Other studies have similarly noted that while both mild and severe cases tend to show peripheral bilateral opacities, ICU patients often have more extensive lung involvement. In 1 comparative analysis, intubated (ICU) patients had a greater number of lung zones affected on X-ray than non-intubated patients (approximately 10–12 zones vs 0–6 zones), despite a similar peripheral distribution in both groups.^[35]^ Radiographic patterns were closely aligned with disease severity and outcomes. Extensive bilateral and peripheral opacities were predominantly observed in ICU-admitted patients and were associated with poorer prognosis. Progression of lung infiltrates generally occurred within 3 to 5 days of ICU admission, while partial resolution was observed among survivors at later follow-up. These findings reinforce the role of chest imaging as an adjunctive marker of severe disease and predictor of adverse outcomes in COVID-19. Taken together, our ICU radiographic findings mirror those reported in the literature for severe COVID-19, characterized by initially peripheral, bilateral infiltrates that become more diffuse and extensive as the disease progresses.

Key strengths of this study include the inclusion of a well-defined hospital cohort with confirmed diagnoses, detailed cytokine profiling, and integration of clinical, radiological, and laboratory data to enable multifactorial analysis. This study adds novel regional evidence by characterizing the clinical, radiological, and inflammatory profiles of COVID-19 patients during the early phase of the pandemic in Saudi Arabia. It integrates demographic and cytokine data within a single analytical framework, highlighting IL-8 and TNF-α as potential markers of disease severity and mortality. Unlike most international cohorts, this analysis captures patients from 3 major hospitals in the Eastern Province, providing a distinctive Middle Eastern perspective on early COVID-19 pathophysiology. These findings contribute to a broader understanding of how inflammatory and demographic factors intersect to drive severe disease outcomes.

However, this study has several important limitations. First, the sample size was relatively small, which may limit the statistical power and generalizability of the findings. Second, there were instances of missing cytokine data for some patients, which could introduce bias or reduce the robustness of our analyses of inflammatory markers. This also might explain why the data for IL-6 was non-significant in logistic regression. Third, as an observational study, there is a possibility of selection, and unmeasured confounders could have influenced who required ICU admission. Additionally, we measured cytokine levels at a single time point without longitudinal follow-up, preventing us from capturing dynamic changes in inflammatory markers over the course of illness or in response to treatments. Another key limitation is the study’s specific timeframe (February–August 2021), which coincided with the circulation of emerging SARS-CoV-2 variants and the initial, rapid vaccine rollout.^[36,37]^ This temporal context defines our cohort as representative of the early variant era, likely including a mix of unvaccinated and partially vaccinated patients, and may limit comparability to later pandemic waves. The absence of individual vaccination status data limits our ability to fully assess its influence on outcomes. These limitations should be considered when interpreting the results, and future studies with larger, more diverse cohorts and serial measurements will be needed to validate and expand upon our findings.

In summary, this multi-center study highlights the distinct clinical, inflammatory, and radiological features of COVID-19 patients during the early variant era in Saudi Arabia. ICU-admitted patients exhibited higher levels of IL-8 and TNF-α, which were strongly associated with mortality, alongside characteristic bilateral and peripheral lung infiltrates on imaging. Routine cytokine profiling, particularly IL-8 and TNF-α, at hospital admission and during early clinical deterioration may aid in risk stratification and timely intervention, while larger longitudinal studies are warranted to validate these biomarkers and assess their role in guiding therapeutic decision-making. Amikacin use was linked to increased mortality, suggesting the need for cautious antimicrobial stewardship in critically ill patients. Collectively, these insights enhance understanding of severe COVID-19 in a Middle Eastern context and emphasize the value of integrating inflammatory and clinical indicators into future pandemic preparedness and management frameworks.

## Author contributions

**Data curation:** Zahraa Alali, Jumana Aljishi, Hani Makky Al Salam, Maryam Alhashim.

**Formal analysis:** Zahraa Alali, Muna Ali Alali.

**Funding acquisition:** Kholoud Alwosaibai.

**Investigation:** Raha Al Dhafir, Fatemah Hussain Benalshaikh, Abdul Salam, Salma A. Aalmri, Kholoud Alwosaibai.

**Methodology:** Muna Ali Alali, Maryam Alhashim.

**Project administration:** Kholoud Alwosaibai.

**Resources:** Kholoud Alwosaibai.

**Validation:** Fatimah Saud Alzaher.

**Writing – original draft:** Zahraa Alali.

**Writing – review & editing:** Zahraa Alali, Jumana Aljishi, Fatimah Saud Alzaher, Raha Al Dhafir, Muna Ali Alali, Fatemah Hussain Benalshaikh, Hani Makky Al Salam, Maryam Alhashim, Abdul Salam, Salma A. Aalmri, Kholoud Alwosaibai.

## Supplementary Material




