# Supplementary material for: Clinical and inflammatory predictors of ICU admission and mortality in COVID-19: A retrospective multi-center cohort study from Saudi Arabia
Source: Medicine (Baltimore). 2026 Jan 2;105(1):e46716. doi: 10.1097/MD.0000000000046716 (PMC12778117; doi:10.1097/MD.0000000000046716)
Supplement: Supplementary file 1 [file medi-105-e46716-s001.docx]

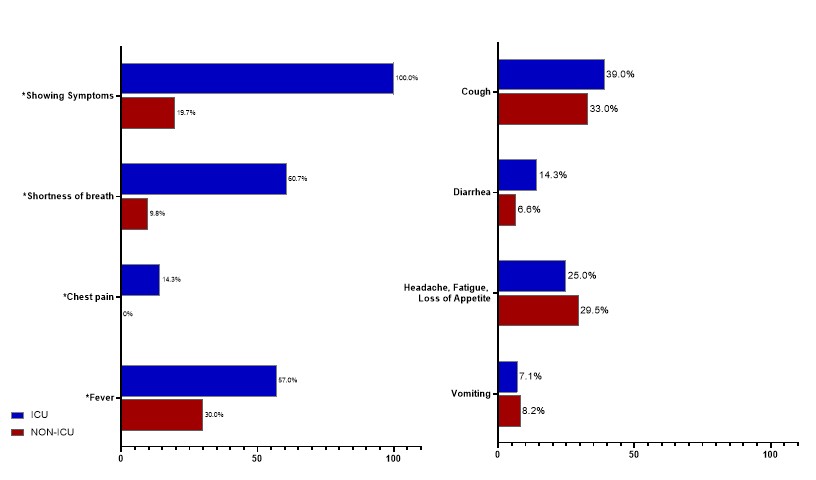


**Supplementary Fig. 1** Comparison of clinical symptoms between ICU and non-ICU COVID-19 patients. The bar graph displays the percentage of patients exhibiting various symptoms, with blue bars representing ICU patients and red bars representing non-ICU patients. Symptoms marked with an asterisk (*) indicate statistically significant differences between the groups, as determined by the Chi-square test (p < 0.05). Symptom categories include showing symptoms, shortness of breath, chest pain, fever, cough, diarrhea, headache, fatigue, loss of appetite, and vomiting.


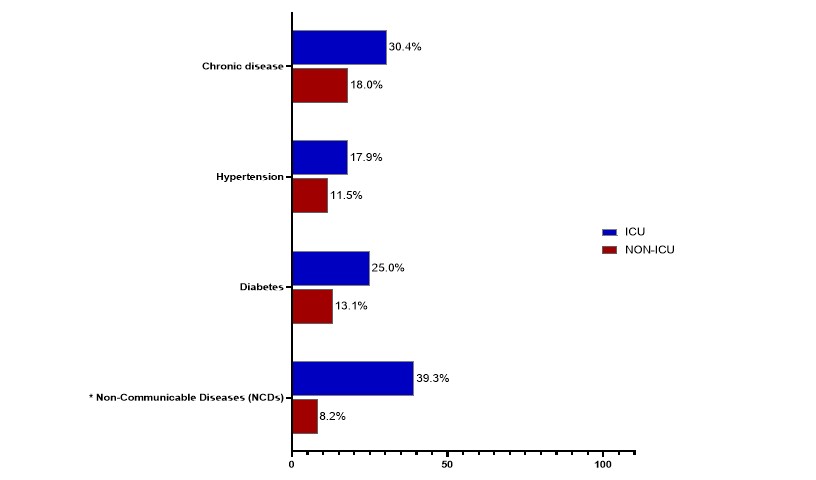


**Supplementary Figure 2.** Prevalence of non-communicable diseases (NCDs) among ICU and non-ICU COVID-19 patients. The graph illustrates the percentage of patients with chronic diseases, hypertension, and diabetes, with blue bars representing ICU patients and red bars representing non-ICU patients. The category marked with an asterisk (*) represents a composite of all non-communicable diseases examined, indicating a statistically significant difference between the groups as determined by the Chi-square test (p < 0.05).
